# PD-1 and PD-L1 Expression on Circulating Lymphocytes as a Marker of Epstein-Barr Virus Reactivation-Associated Proliferative Glomerulonephritis

**DOI:** 10.3390/ijms21218001

**Published:** 2020-10-27

**Authors:** Ewelina Grywalska, Iwona Smarz-Widelska, Izabela Korona-Głowniak, Sebastian Mertowski, Krzysztof Gosik, Anna Hymos, Jarosław Ludian, Paulina Niedźwiedzka-Rystwej, Jacek Roliński, Wojciech Załuska

**Affiliations:** 1 Department of Clinical Immunology and Immunotherapy, Medical University of Lublin, 20-093 Lublin, Poland; mertowskisebastian@gmail.com (S.M.); krzysiekgoja@gmail.com (K.G.); jaroslaw.ludian@umlub.pl (J.L.); jacek.rolinski@gmail.com (J.R.); 2 Department of Nephrology, Cardinal Stefan Wyszynski Provincial Hospital in Lublin, 20-718 Lublin, Poland; i.widelska@interia.pl; 3 Department of Pharmaceutical Microbiology, Medical University of Lublin, 20-093 Lublin, Poland; iza.glowniak@umlub.pl; 4 Department of Otolaryngology and Laryngeal Oncology, Medical University of Lublin, 20-954 Lublin, Poland; annahymos@gmail.com; 5 Institute of Biology, University of Szczecin, 71-412 Szczecin, Poland; paulina.niedzwiedzka-rystwej@usz.edu.pl; 6 Department of Nephrology, Medical University of Lublin, 20-954 Lublin, Poland; wojciech.zaluska@umlub.pl

**Keywords:** Epstein–Barr virus, EBV, glomerulonephritis, immunology, CD4+ T lymphocyte, CD8+ T lymphocyte, CD19+ B lymphocyte, programmed cell death protein 1, PD-1, programmed death-ligand 1, PD-L1

## Abstract

Alterations to the programmed cell death protein-1 (PD-1) pathway were previously shown to be involved in a poorer prognosis for patients with proliferative glomerulonephritis (PGN). Here, we investigated the association between several infectious agents and the expression of PD-1 and its ligand (PD-L1) on T and B lymphocytes in patients with PGN and nonproliferative glomerulonephritis (NPGN). A cohort of 45 newly-diagnosed patients (23 with PGN and 22 with NPGN) and 20 healthy volunteers was enrolled. The percentage of peripheral blood mononuclear cells expressing PD-1 and PD-L1 antigens was determined by flow cytometry. We found PD-1 and PD-L1 expression on T and B lymphocytes was higher in PGN patients than in NPGN patients and controls. We also found that reactivation of the Epstein-Barr virus (EBV) correlated with the expression of PD-1/PD-L1 antigens in patients with PGN. Further receiver operating characteristic analysis indicated that PD-1 expression could distinguish EBV-positive PGN patients from those with NPGN or healthy controls. The use of PD-1 expression as a non-invasive marker of PGN should be further investigated.

## 1. Introduction

Programmed cell death protein 1 (also known as PD-1 and CD279) is a cell surface receptor that in humans is expressed on T cells, B cells, monocytes/macrophages, as well as some cancer cells, such as melanoma and liver cancer [1,2,3,4]. PD-1 functions as an immune checkpoint and helps to prevent overactivation of T cells that may lead to autoimmunity [5]. Studies have shown that T cells with high PD-1 expression lose the ability to eliminate cancer and infectious agents [6,7,8]. It is also worth mentioning that most data in databases is related to mRNA expression profiles and not PD-1 protein on the cell surface, considering that expression of PD-1 in the tumor tissues may at least in part derive from T cells infiltrating tumors [9]. PD-1 can also regulate T cell exhaustion, which is a state of T cell dysfunction [10]. Levels of PD-1 on T cells change depending on the immune activation status. While PD-1 is virtually absent on naïve T cells, its expression is upregulated following T-cell receptor (TCR)-mediated activation and is readily observed on both activated and exhausted T cells [5]. Upon ligation of PD-1 to its ligands (PD-L1 and PD-L2), the complex binds to the TCR, which in turn inhibits the phosphorylation of the CD3-γ chain [11,12]. As a result, TCR signaling during antigen presentation to naïve T cells is inhibited [13]. Therefore, in the case of persistent auto- or exo-antigenic stimulation, the PD-1/PD-L1/2 pathway plays a crucial inhibitory role [14,15,16].

Glomerulonephritis (GN) is an immune-mediated disorder that often develops into severe kidney failure [17]. The immune response to foreign antigens, such as those derived from microorganisms, may lead to the creation of antibody complexes and their deposition in the kidneys. Such deposits trigger inflammation, which results in progressive impairment of the kidney function. Although B cells were first mentioned as involved in GN pathogenesis, it has become clear that also T cells and other immune cells, such as dendritic cells and macrophages, play an important role in the development of GN. CD8+ and CD4+ T cells have been found in kidney biopsies taken from the GN patients [18,19]. 

Proliferative glomerulonephritis (PGN) is characterized by an increased number of cells in the glomeruli, while in nonproliferative glomerulonephritis (NPGN), the number of cells does not change [20]. In both these diseases, inflammation causes increased protein permeability in the cells surrounding the glomeruli, leading to elevated protein excretion in the urine (proteinuria) [21]. However, the disease course markedly differs between PGN and NPGN. NPGN is typically associated with nephrotic syndrome (characterized by hypoalbuminemia and hyperlipidemia in addition to proteinuria, and has a good prognosis) [22,23]. Meanwhile, PGN causes nephritic syndrome (which is characterized by blood in the urine and decreased urine production and can rapidly lead to end-stage renal failure) [24,25]. It is crucial to differentiate between these two types of GN to choose the appropriate treatment. However, currently, the distinction between PGN and NPGN requires a kidney biopsy [26].

Although the immunopathogenesis of these disorders is not fully understood, many researchers have observed a link between infections and the development of primary GN [27,28,29]. Moreover, in a cohort of 20 patients with primary GN, we previously showed that the frequencies of PD-1-positive and PD-L1-positive T and B lymphocytes were higher among patients suffering from PGN than in those diagnosed with NPGN or in the control group [30]. Therefore, in the current study, we aimed to examine a potential involvement of infectious agents in the upregulation of the PD-1/PD-L1 axis in patients with primary GN and to determine whether these proteins could serve as non-invasive markers to distinguish PGN from NPGN.

## 2. Results

### 2.1. Characteristics of Basic Parameters in Patients and Healthy Controls

The baseline characteristics of patients and healthy controls included in the study are summarized in Table 1.

Table 1 presents the characteristics of complete blood counts, kidney function parameters, proteinograms, and complement components in patients and healthy controls.

Table 2 presents basic characteristics of NPGN and PGN patients.

### 2.2. PGN Patients Have a More “Exhausted” Lymphocyte Profile than NPGN Patients and Healthy Controls

Peripheral blood mononuclear cells (PBMCs) isolated from patients suffering from primary GN were immunophenotyped and compared to an age- and sex-matched control group. We found no significant changes in the frequencies of T cells, B cells, and CD4+ T cells between the two groups (Table 3). However, several immune cell populations, including NK cells and CD8+ T lymphocytes, were decreased in patients with primary GN compared to the healthy controls. Interestingly, the frequencies of immune cells expressing either PD-1 or PD-L1 were significantly increased in the PN patients as compared to controls.

We have also compared basic lymphocyte subsets frequencies and the percentages of lymphocytes expressing PD-1 and PD-L1 molecules between NPGN and PGN patients. Our analyses revealed that PGN patients were characterized by higher PD-1 and PD-L1-positive T CD4+ cells as well as PD-1-positive T CD8+ and B CD19+ cells (Table 4).

### 2.3. Understanding the Phenomenon of Lymphocyte Exhaustion in PGN Patients and NPGN Patients

To further investigate the reason behind the high percentages of PD-1 and PD-L1 lymphocytes, we looked for the presence of infectious agents, including standard bacterial and fungal pathogens and several viruses. All tests came back negative, except for the Epstein-Barr virus (EBV): 60.87% of patients with PGN were positive (*n* = 14; 10 patients with membranoproliferative GN and four patients with IgA nephropathy). Furthermore, we found EBV reactivation markers only in the PGN group, which together indicates that those patients were actively infected with EBV due to viral reactivation.

The PCR-based test revealed the presence of EBV DNA copies in patients with PGN: 130.79 (31.68–418.33) copy number/µg DNA in 10 out of 11 patients with membranoproliferative GN and 20.65 (16.33–25.65) copy number/µg DNA in 4 out of 12 patients with IgA nephropathy. Interestingly, analysis of the serological status of the participants revealed that all PGN patients with positive EBV DNA results had antibody titers indicating a viral reactivation, assessed as described by De Paschale et al. [31]. They tested positive for anti-viral capsid antigen (VCA) IgG, IgM, and IgA; anti-EBNA-1 IgG and IgA; and anti-early antigen (EA) IgA antibodies. On the other hand, patients with primary GN that were negative for EBV DNA and all healthy controls had the anti-EBV status suggesting an EBV reactivation [31]; positive only for anti-VCA IgG and anti-EBNA-1 IgG. All groups were negative for anti-EBNA-1 IgM and anti-EA IgM and IgG.

### 2.4. Correlation of PD-1 and PD-L1—Positive Cells and EBV DNA Copy Numbers

**Figure 1:**
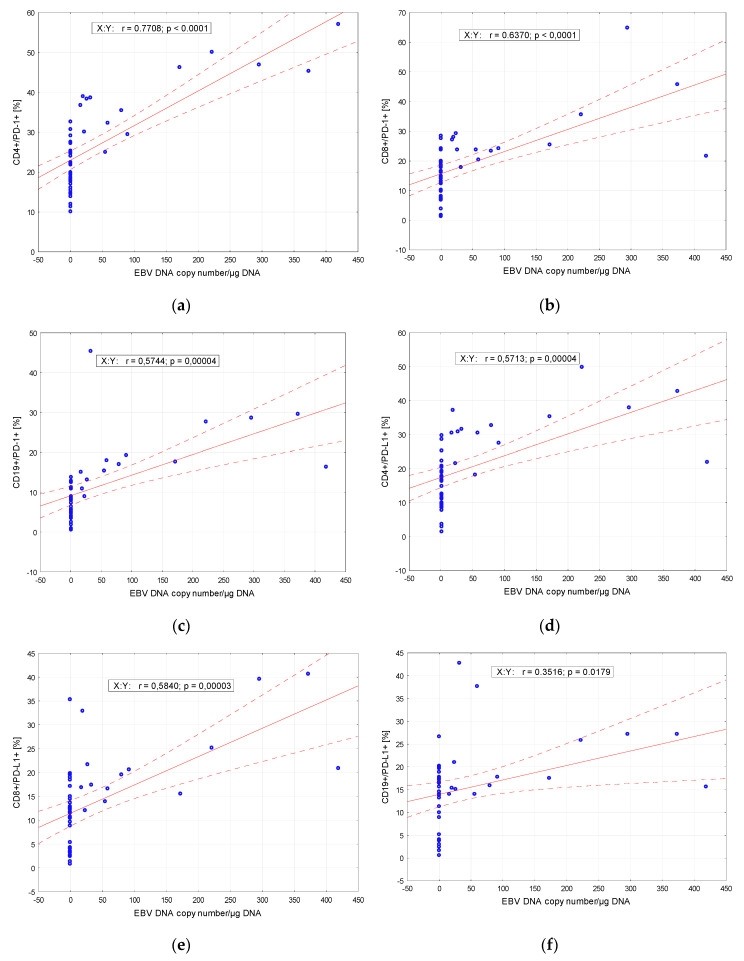
Correlation of PD-1 and PD-L1 – positive cells and Epstein-Barr virus (EBV) DNA copy numbers: (**a**) CD4+/PD-1 (%); (**b**) CD8+/PD-1 (%); (**c**) CD19+/PD-1 (%); (**d**) CD4+/PD-L1 (%); (**e**) CD8+/PD-L1 (%); (**f**) CD19+/PD-L1 (%).

Considering a possible correlation between the EBV DNA copy number and the percentages of lymphocytes expressing PD-1 and PD-L1 molecules, a statistical analysis was applied, indicating their high positive correlations in all tested lymphocytes with the highest one for CD4+/PD-1 positive lymphocytes (Figure 1).

### 2.5. ROC Analyses of PD-1 and PD-L1—Positive Cells as Possible Non-Invasive Markers to Distinguish Type of Primary Glomerulonephritis

**Figure 2:**
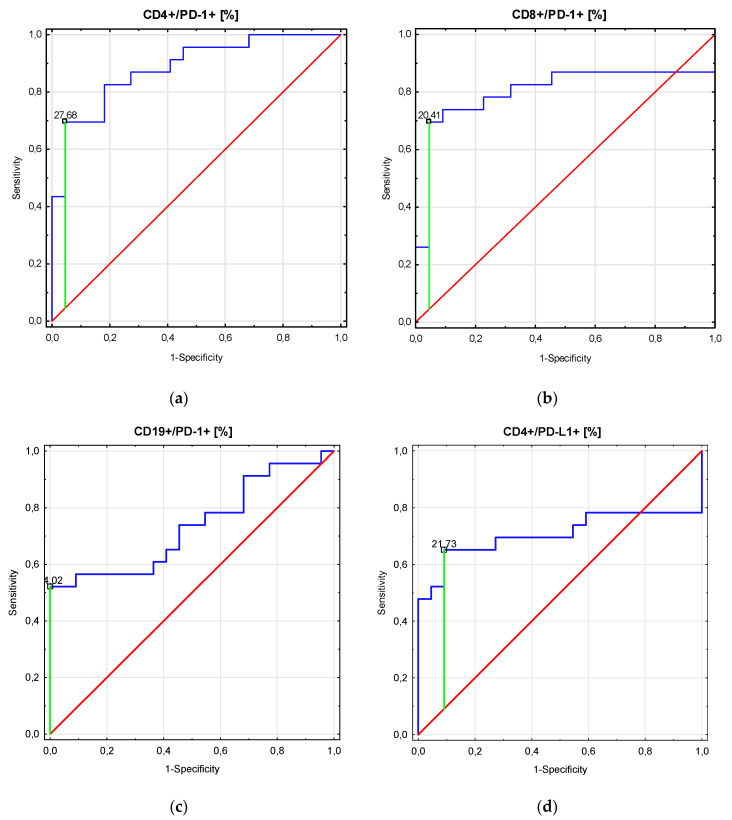
Receiver operating curve (ROC) analysis to determine diagnostic accuracy in differentiation of patients with proliferative glomerulonephritis (PGN). (**a**) CD4+/PD-1 (%); (**b**) CD8+/PD-1 (%); (**c**) CD19+/PD-1 (%); (**d**) CD4+/PD-L1 (%).

Figure 2 and Table 5 demonstrate the receiver operating characteristic (ROC) analysis for the six immunological parameters. As the area under the curve (AUC) shows, the percentages of CD4+/PD-1+ and CD8+/PD-1+ cells were the most sensitive and specific parameters to determine patients with PGN (AUC = 0.89 and 0.80, respectively).

## 3. Discussion

In most countries, primary GN presents about 20% of chronic kidney diseases. It is more persistent in young people, representing the most frequent cause of end-stage renal disease. Moreover, unlike major causes of chronic kidney disease such as diabetes and hypertension, many young adults carry a lifelong burden of chronic kidney disease [32]. Primary GN is a heterogeneous group of diseases, qualified as orphan diseases, and not many clinical trials have been performed to explore the condition mechanisms and potential therapies. The most recent guidelines were prepared in 2012 by the Kidney Disease: Improving Global Outcomes (KDIGO) initiative on the treatment of glomerular diseases [32]. Since no specific clinical signs of primary GN exist, an early and accurate diagnosis is challenging. The clinical course of primary GN is variable, differing from patients presenting with conditions such as hypertension, proteinuria, hematuria, and raised serum creatinine concentrations, through those with a massive weight gain and edema with nephrotic syndrome, to patients rapidly progressing and presenting uremia [33].

It is agreed, that a kidney biopsy is the diagnostic tool of choice for GN as far as the procedure is well controlled and follows the standards, i.e., it is performed by an experienced physician, the patient has no coagulation disorders, normal blood pressure, no urinary tract infections, no antiplatelet drugs were taken for seven days prior to biopsy, and the biopsy is carried out under the ultrasound view with a 16–18 gauge needle) [34]. Then, perirenal hematoma is distinguished in 50–80% of patients and arteriovenous fistulas are present in about 15% of patients [35]. Bleeding in which transfusion, surgical intervention, or nephrectomy is necessary, occurs in less than 0.5% of cases. Since complications may appear up to 8h after the procedure, an overnight hospitalization is required after a kidney biopsy, particularly if moderate-to-advanced chronic kidney disease occurs [35]. Therefore, there is an urgent need for identification of novel, non-invasive markers to diagnose primary GN [25,30]. Our study suggest that PD-1 and PD-L1 antigen expression on the surface of circulating lymphocytes may be a sensitive marker of PGN.

Recent studies have shown that viral infections may play a pivotal role in the development of secondary GN as well as PGN, such as IgA nephropathy or membranoproliferative GN [36,37,38,39]. To this end, our studies demonstrate the association of the presence of EBV DNA copies in the peripheral blood with the expression of PD-1 and PD-L1 antigens on the surface of circulating lymphocytes.

It is worth noticing, that EBV is enumerated among human viruses with a proven role in oncogenesis, and the development of autoimmune disorders, as well as immunosuppression [40,41,42,43]. The life cycle of EBV is particularly complex and the virus undergoes the latency phase during which it expresses several genes impacting oncogenesis [44,45,46,47]. Recent studies have shown that EBV DNA is present in large quantities in patients with autoimmune diseases of several etiologies [48,49]. However, there are no proofs on the effect of EBV reactivation on the expression of PD-1 and PD-L1 antigens in patients with primary GN. To our best knowledge, our study is the first one to show that patients with PGN have detectable levels of EBV DNA and a serologic profile specific for EBV reactivation. Moreover, higher numbers of EBV DNA copies were noted in patients with membranoproliferative GN comparing with patients with IgA nephropathy. A possible cause for the above may be due to the viral inhibiting mechanisms aimed at the elimination or enhancement of expression of tolerogenic molecules, and ineffective antigen presentation. Glomerular cells in PGN are not properly recognized by the immune system of the patients. One of the reasons for this may be an EBV-induced defective signal transduction by the PD-1/PD-L1 axis as it has already been described in other disorders [40,41,43,50]. It is likely that in people with a specific immune profile, inappropriate recognition of EBV antigens induces the production of specific proteins, which in the process of antigenic mimicry interact with the components of the immune system, leading to the development of GN. It has been previously demonstrated that EBV antigens were present in the glomeruli of patients with GN and the number of DNA EBV copies correlated with the degree of glomerular injury [51]. It is known that in some patients with GN, EBV-associated lymphoma may occur years after diagnosis [52]. Similarly, a coexistence of a nephrotic syndrome and infectious mononucleosis has been also reported and may confirm the hypothesis of the involvement of EBV in the induction of GN [53]. Furthermore, it has been demonstrated in the animal model and confirmed in our study that EBV infection, diagnosed based on detection of viral DNA, can lead to the development of GN [54]. Thus, our results suggest that membranoproliferative GN may be considered as an EBV-associated disease. Determining the relation between EBV reactivation and upregulation of PD-1/PD-L1 axis may change the perception of the etiopathogenesis of heterogenic course of the disease with the further impact on the therapeutic approach to membranoproliferative GN and EBV-associated cases of IgA nephropathy.

### Limitations of the Study

The study included a cautiously evaluated group of newly diagnosed NPGN and PGN patients with strict inclusion criteria, including lack of any infection three months prior to the study, no immunomodulatory treatment, no allergy, etc. Due to the fact that primary GN in adult patients is a rare disease, we only found 45 patients fulfilling the criteria, and we thoroughly analyzed samples obtained from those patients and compared them with other clinical parameters. A larger study group could deliver more statistically significant correlations or differences between patients with PGN and healthy controls. Additionally, longitudinal studies on how the kidney function of the study participants changes over time and whether the progression towards higher classes of end-stage renal disorder correlates with the PD-1/PD-L1 expression and EBV loads in the patients would deliver further clinically important information. Indeed, we plan to follow the patients described in this study and add the new data on how their clinical status changes and if it is reflected by the changes in the PD-1/PD-L1 expression and EBV loads.

## 4. Materials and Methods

### 4.1. Patients and Controls

This study included 45 newly-diagnosed, treatment-naïve patients with primary GN (28 men and 17 women). Twenty healthy volunteers (ten men and ten women) served as a control group. Exclusion criteria were the use of immunomodulating agents or hormonal preparations, or any signs of infection for at least three months prior to the study. In addition, persons who underwent a blood transfusion, presented with an autoimmune condition or allergy, had a history of oncological therapy or prior treatment for tuberculosis or other chronic conditions that could be associated with impaired cellular or humoral immunity were excluded from the study.

This study was approved by the Ethics Committee of the Medical University of Lublin (Decision No. KE-0254/290/2014) and was conducted in accordance with the Helsinki Declaration. All patients and volunteers signed an informed consent form before blood collection.

The diagnosis of GN was made by histological analysis of renal biopsy samples, including standard hematoxylin-eosin staining and immunohistochemical staining for immune complexes, in accordance with standard criteria [31] and previously published methodology [33]. PGN (including IgA nephropathy and membranoproliferative GN) and NPGN (including membranous GN and minimal-change disease) were differentiated based on proliferative changes in the glomeruli [31]. All patients underwent complete blood count, kidney function assessment, and diagnosis for infectious agents, which was performed in the certified diagnostic laboratories of the Independent Public Clinical Hospital No. 4 in Lublin and the Provincial Specialist Hospital in Lublin. The characteristics of the study and control groups are presented in Table 6.

### 4.2. Preparation of Material

A total of 15 mL of peripheral blood was collected in EDTA-coated tubes (Sarstedt, Nümbrecht, Germany), and PBMCs and plasma were isolated by density gradient centrifugation (using Ficoll-Paque™; Milteny Biotec, Bergisch-Gladbach, Germany) at 400 g for 30 min. Plasma was stored at −80 °C until use. Only PBMCs with a viability of ≥95% (determined using 0.4% trypan blue solution; Sigma Aldrich, Hamburg, Germany) were used in subsequent experiments [25,30].

### 4.3. Immunophenotyping

Immunophenotyping was performed using a FACSCalibur flow cytometer (BD Biosciences, San Jose, CA, USA) equipped with a 488 nm argon laser, as previously described [30,40,43]. Briefly, cells were stained with monoclonal antibodies (purchased from BD Biosciences, San Jose, CA, USA) conjugated with different fluorochromes to determine the proportion of the specific cell types and the surface expression of PD-1 and PD-L1. Percentages of PD-1-positive and PD-L1-positive T and B lymphocytes were determined using combinations of the following monoclonal antibodies: CD45/FITC, CD14/PE, CD3/CyChrome, CD19/FITC, CD4/FITC, CD8/FITC, CD279 (PD-1)/PE, and CD274 (PD-L1)/PE. A minimum of 10,000 events was acquired. We also determined the percentages of cells expressing specific surface markers. All FACS data were analyzed using the CellQuest Software (BD Biosciences, San Jose, CA, USA).

### 4.4. Patients’ Infection Status Assessment

All samples were tested for the presence of common bacterial, viral, and fungal pathogens. No bacterial (aerobic and non-aerobic) or fungal pathogens were identified in any samples via standard culturing methods. Moreover, using qualitative PCR-based tests used in certified hospital diagnostic laboratory, we did not identify any genetic material of the hepatitis A virus (HAV), hepatitis B virus (HBV), hepatitis C virus (HCV), hepatitis D virus (HDV), hepatitis E virus (HEV), enteroviruses, adenoviruses, human immunodeficiency virus (HIV), *Herpes simplex* virus 1 and 2 (HSV-1 and -2), cytomegalovirus (CMV), human papillomavirus (HPV), parvovirus B19, influenza virus, *Borrelia burgdorferi*, *Chlamydia trachomatis*, *Chlamydia pneumoniae*, *Mycobacterium tuberculosis*, *Toxoplasma gondii*, *Ureaplasma* spp., or *Listeria* spp. Surprisingly, significant counts of EBV DNA copies were found in 14 PGN patients [41]. We also performed serological tests separately for each patient and each of the following pathogens: severe acute respiratory syndrome coronavirus 2 (SARS-CoV-2), HCV, CMV, EBV, *Toxoplasma gondii*, as well as anti-streptolysin O titers [42].

### 4.5. DNA Isolation and Calculation of EBV Load and Assessment of Anti-EBV Antibody Status

DNA was isolated from five million PBMCs with the QIAamp DNA Blood Mini Kit (QIAGEN, Hilden, Germany), as per manufacturer’s instructions, and the number of EBV-specific DNA copies was calculated with the ISEX variant of the EBV PCR kit (GeneProof, Brno, Czech Republic). The concentration and purity of the isolated DNA were verified with a BioSpec-nano spectrophotometer (Shimadzu, Kyoto, Japan). All samples were analyzed in duplicate, and a negative control (DNA elution buffer) was included. A specific conservative DNA sequence for the EBV nuclear antigen 1 (EBNA-1) gene was amplified using a 7300 Real Time PCR System (Applied Biosystems, Foster City, CA, USA). The number of viral DNA copies/μL of eluent was adjusted for the efficiency of DNA isolation and expressed as the viral DNA copy number/μg of DNA. Due to the detection threshold of ten EBV DNA copies/μL, all samples below this threshold were considered EBV negative. Anti-EBV serological status was assessed using ELISA kits (IBL International, Hamburg, Germany) on the Victor TM3 (PerkinElmer, Waltham, MA, USA). IgA, IgM, and IgG antibodies recognizing the early antigen (EA), viral capsid antigen (VCA), and EBNA-1 were measured, and manufacturer-specified cut-offs applied [40,43].

### 4.6. Statistical Analysis

The statistical analysis was carried out with Tibco Statistica 13.3 (StatSoft, Palo Alto, CA, USA). The normal distribution of continuous variables was tested using the Shapiro–Wilk test. The values of the parameters were presented as arithmetic means and their standard deviations (SD) for normally distributed data and as medians, minimum, and maximum values for non-normal data. Student’s t-test was used for independent variables and the Mann–Whitney U-test as an intergroup comparison component. Kruskal–Wallis ANOVA and multiple comparisons of mean ranks (as a post-hoc analysis) were applied for the analysis of differences between more than two groups. The power and direction of the association between pairs of continuous variables (studied groups) were determined using Spearman’s coefficient of rank correlation. The distributions of discrete variables in groups were compared with Pearson’s chi-square test or Fisher’s exact test. Additionally, the diagnostic effectiveness of the laboratory test was determined using receiver operating characteristic (ROC) curves for parameters related to the different groups of patients. Areas under the ROC curves (AUCs) were calculated for each parameter and compared. The error was set at 5% and significance at *p*-value < 0.05 [25].

## 5. Conclusions

We speculate that reactivation of EBV may occur in patients with PGN due to an imbalance in the immune system. In particular, the loss of immune homeostasis in PGN patients increases their susceptibility to EBV reactivation, which leads to even more severe immune system dysregulation and aggravation of PGN symptoms. However, whether infection with EBV predisposes people to develop PGN requires further investigation. Nevertheless, we observed that EBV positive status has been accompanied by an increase in PD-1 expression on the immune cells and that such a combination of markers was characteristic for PGN patients. Therefore, we propose that PD-1 expression level and EBV reactivation status could be used in the future as non-invasive markers of GN classification.

## Figures and Tables

**Table 1:** Results of the complete blood counts, kidney function parameters, proteinograms, and complement components in patients and healthy controls.

Parameters	Study Group (*n* = 45)		Control Goup (*n* = 20)		t/Z	*p* Value
	Mean ± SD	Median (Range)	Mean ± SD	Median (Range)		
WBC [10^3^/mm^3^]	6.8 ± 1.7	6.6 (4.3–9.9)	6.8 ± 0.4	6.7 (6.3–7.6)	−0.55	0.58
LYM [10^3^/mm^3^]	2.1 ± 0.7	1.96 (1.2–3.7)	2.5 ± 0.6	2.5 (1.5–3.7)	−2.53	0.011
RBC [10^6^/mm_3_]	4.7 ± 1.6	4.4 (3.3–11.8)	5.2 ± 0.4	5.12 (4.5–5.8)	−4.7	<0.0001
HGB [g/dL]	13.2 ± 1.6	13.2 (9.3–16.4)	14.3 ± 1.2	14.35 (12.5–16.9)	−2.85	0.0058
PLT [10^3^/mm^3^]	238.9 ± 61.3	222.0 (147.0–410.0)	279.0 ± 57.0	281.5 (186.0–403.0)	−2.49	0.013
Urea [mg/dL]	51.9 ± 28.6	44.3 (13.0–115.7)	31.4 ± 6.9	32.0 (18.0–42.0)	2.59	0.0097
BUN [mg/dL]	24.3 ± 13.4	20.7 (6.1–54.0)	14.7 ± 3.2	14.95 (8.4–19.6)	2.59	0.0097
Serum creatinine [mg/dL]	1.2 ± 0.6	0.96 (0.37–2.3)	0.9 ± 0.1	0.9 (0.7–1.1)	1.07	0.29
eGRF [ml/min/1.73 m^2^]	86.1 ± 33.1	81.5 (26.7–146.8)	125.6 ± 10.3	121.1 (115.0–148.2)	−5.2	<0.0001
Serum uric acid [mg/dL]	6.9 ± 1.7	7.1 (3.8–11.9)	6.2 ± 1.4	6.95 (3.7–7.9)	1.73	0.083
Total quantity of protein in a 24-h urine collection test [g/24 h]	5.7 ± 5.6	4.8 (0.01–24.9)	0.0 ± 0	0.0	6.39	<0.0001
Serum IgG [g/L]	6.6 ± 2.8	6.3 (2.8–15.7)	12.7 ± 1.4	12.79 (10.1–15.5)	−5.79	<0.0001
Serum IgM [g/L]	1.7 ± 1.1	1.2 (0.2–3.98)	1.7 ± 0.3	1.6 (1.2–2.2)	−1.02	0.31
Serum IgA [g/L]	2.4 ± 1.6	2.0 (0.3–6.1)	2.4 ± 0.8	2.56 (0.9–3.9)	−0.76	0.45
Serum total protein [g/dL]	5.4 ± 1.0	5.4 (3.2–7.4)	7.4 ± 0.6	7.35 (6.4–8.2)	−8.0	<0.0001
Serum albumin [g/L]	2.6 ± 0.9	2.8 (0.6–4.1)	4.2 ± 0.4	4.2 (3.5–4.75)	−7.75	<0.0001
Serum complement component C3 [g/L]	1.2 ± 0.3	1.2 (0.4–2.0)	1.3 ± 0.2	1.2 (0.95–1.8)	−0.85	0.40
Serum complement component C4 [g/L]	0.3 ± 0.08	0.28 (0.11–0.55)	0.3 ± 0.08	0.3 (0.15–0.4)	0.82	0.42

SD: standard deviation; WBC: white blood cells; LYM: lymphocytes; RBC: red blood cells; HGB: hemoglobin; PLT: platelets; BUN: blood urea nitrogen; eGFR: estimated glomerural filtration rate; Ig: immunoglobulin; t/Z: Student’s t-distribution/Z value.

**Table 2:** Basic characteristics of NPGN and PGN patients.

Parameters	NPGN (*n* = 22)		PGN (*n* = 23)		t/Z	*p* Value
	Mean ± SD	Median (Range)	Mean ± SD	Median (Range)		
Male/Female, *n* (%)	14 (63.6)/8 (36.4)		14 (60.9)/9 (39.1)			1.0
Age [years]	42.8 ± 13.2	37.4 (19.0–70.0)	37.4 ± 14.3	35.0 (20.0–70.0)	1.31	0.20
Arterial hypertension, *n* (%)	2 (9.1)		13 (56.5)			0.0012
Urea [mg/dL]	55.2 ± 31.5	45.1 (17.8–115.7)	48.8 ± 25.9	44.3 (13.0–115.7)	0.74	0.46
BUN [mg/dL]	25.8 ± 14.7	21.1 (8.3–54.0)	22.8 ± 12.1	20.7 (6.1–54.0)	0.74	0.46
Serum creatinine [mg/dL]	1.1 ± 0.6	0.9 (0.37–2.3)	1.3 ± 0.6	1.2 (0.5–2.3)	−0.9	0.37
eGRF [ml/min/1.73 m^2^]	106. ± 28.2	115.7 (54.6–146.8)	66.7 ± 25.1	69.9 (26.7–117.3)	4.99	<0.0001
Serum uric acid [mg/dL]	6.6 ± 1.9	6.4 (3.8–11.9)	7.2 ± 1.5	7.6 (4.0–9.3)	−1.19	0.24
Serum IgG [g/L]	6.5 ± 2.1	6.7 (3.2–11.4)	6.7 ± 3.4	6.1 (8.8–15.7)	−0.25	0.80
Serum IgM [g/L]	2.2 ± 1.1	2.7 (0.4–3.98)	1.1 ± 0.7	1.1 (0.2–3.2)	3.87	0.0004
Serum IgA [g/L]	2.0 ± 0.95	2.1 (0.6–3.6)	2.6 ± 1.9	1.9 (0.3–6.1)	−1.34	0.19
Serum total protein [g/dL]	5.4 ± 1.0	5.2 (4.0–7.4)	5.5 ± 1.0	5.7 (3.2–6.9)	−0.57	0.57
Serum albumin [g/L]	2.5 ± 0.8	2.6 (0.8–3.8)	2.8 ± 0.9	2.8 (0.6–4.1)	−0.86	0.40
Total quantity of protein in a 24-hour urine collection test [g/24 h]	8.5 ± 6.1	6.3 (3.0–24.9)	3.0 ± 3.6	0.9 (0.01–10.3)	3.46	0.0005
Serum complement component C3 [g/L]	1.4 ± 0.3	1.3 (0.9–2.0)	1.1 ± 0.3	1.2 (0.4–1.7)	3.29	0.002
Serum complement component C4 [g/L]	0.32 ± 0.1	0.28 (0.2–0.55)	0.3 ± 0.07	0.3 (0.1–0.5)	1.32	0.19

SD: standard deviation; BUN: blood urea nitrogen; eGFR: estimated glomerural filtration rate; Ig: immunoglobulin; NPGN: nonproliferative glomerulonephritis; PGN: proliferative glomerulonephritis; t/Z: Student’s t-distribution/Z value.

**Table 3:** Immunophenotyping of peripheral blood lymphocytes. Frequencies of subpopulations within the leukocyte (CD45+) population are shown.

Variable (Unit)	Study Group (*n* = 45)	Control Goup (*n* = 20)	t/Z	*p* Value
	Median (Range)	Median (Range)		
Frequencies of T CD3+ lymphocytes (%)	74.4 (5.2–87.98)	72.4 (70.0–74.75)	1.36	0.17
Frequencies of B CD19+ lymphocytes (%)	10.98 (1.7–70.3)	10.8 (6.0–14.5)	0.23	0.82
Frequencies of NK cells (%)	12.6 (1.9–36.8)	14.5 (12.6–19.8)	−2.42	0.016
Frequencies of T CD3+/CD4+ cells (%)	43.1 (26.1–63.4)	41.5 (40.5–44.2)	0.68	0.50
Frequencies of T CD3+/CD8+ cells (%)	28.0 (18.1–48.7)	29.97 (28.9–33.2)	−2.30	0.022
T CD3+/CD4+: T CD3+/CD8+ ratio	1.6 (0.7–3.2)	1.4 (1.2–1.5)	1.73	0.084
CD4+/PD-1+ (%)	24.7 (10.3–57.3)	5.3 (2.65–7.7)	6.39	<0.0001
CD8+/PD-1+ (%)	18.0 (1.5–64.8)	3.7 (1.4–6.2)	5.55	<0.0001
CD19+/PD-1+ (%)	10.8 (0.6–45.6)	1.9 (0.4–3.4)	5.31	<0.0001
CD4+/PD-L1+ (%)	17.8 (1.4–49.8)	1.7 (1.0–4.5)	6.16	<0.0001
CD8+/PD-L1+ (%)	12.8 (0.9–40.7)	0.4 (0.3–0.7)	6.39	<0.0001
CD19+/PD-L1+ (%)	15.7 (0.7–43.0)	0.2 (0.07–1.0)	6.37	<0.0001

CD: cluster of differentiation; NK: natural killer; PD-1: programmed cell death protein 1; PD-L1: programmed death-ligand 1; t/Z: Student’s t-distribution/Z value.

**Table 4:** Results of the immunophenotyping of the peripheral blood lymphocytes of the NPGN and PGN patients. Cells were enumerated and presented as % of a specific subpopulation within the leukocyte (CD45+) population.

Variable (Unit)	NPGN (*n* = 22)	PGN (*n* = 23)	t/Z	*p* Value
	Median (Range)	Median (Range)		
Frequencies of T CD3+ lymphocytes (%)	76.9 (5.2–87.98)	73.3 (63.3–85.1)	−0.32	0.75
Frequencies of B CD19+ lymphocytes (%)	9.5 (1.7–70.3)	11.3 (7.0–19.1)	0.19	0.85
Frequencies of NK cells (%)	10.2 (1.9–36.8)	12.9 (7.3–35.3)	−0.76	0.89
Frequencies of T CD3+/CD4+ cells (%)	44.7 (26.1–63.4)	41.4 (34.5–53.3)	1.06	0.45
Frequencies of T CD3+/CD8+ cells (%)	27.3 (18.4–48.7)	28.9 (18.1–42.1)	−0.40	0.77
T CD3+/CD4+: T CD3+/CD8+ ratio	1.6 (0.7–3.2)	1.4 (0.8–2.7)	0.73	0.47
CD4+/PD-1+ (%)	18.6 (10.3–32.6)	30.8 (16.3–57.3)	−5.38	<0.0001
CD8+/PD-1+ (%)	13.2 (4.0–27.8)	23.9 (1.5–64.28)	−3.22	0.0025
CD19+/PD-1+ (%)	7.9 (0.6–14.0)	14.0 (0.9–45.6)	−2.99	0.0046
CD4+/PD-L1+ (%)	16.7 (9.0–28.7)	27.7 (1.4–49.8)	−2.74	0.0088
CD8+/PD-L1+ (%)	12.1 (1.4–35.5)	16.8 (0.9–40.7)	−1.39	0.17
CD19+/PD-L1+ (%)	14.4 (1.7–26.8)	15.9 (0.7–43.0)	−1.56	0.13

CD: cluster of differentiation; NK: natural killer; PD-1: programmed cell death protein 1; PD-L1: programmed death-ligand 1; NPGN: nonproliferative glomerulonephritis; PGN: proliferative glomerulonephritis; t/Z: Student’s t-distribution/Z value.

**Table 5:** Receiver operating characteristic (ROC) analysis to determine diagnostic accuracy in the differentiation of patients with proliferative glomerulonephritis (PGN). AUC: area under the curve.

Parameter	Prognostic Value	AUC	95% CI	*p* Value
CD4+/PD-1+ (%)	27.68	0.89	0.79–0.98	<0.0001
CD8+/PD-1+ (%)	20.4	0.80	0.66–0.95	<0.0001
CD19+/PD-1+ (%)	14.2	0.74	0.59–0.88	0.002
CD4+/PD-L1+ (%)	21.73	0.70	0.54–0.88	0.017
CD8+/PD-L1+ (%)	15.51	0.62	0.45–0.79	0.15
CD19+/PD-L1+ (%)	20.27	0.62	0.45–0.78	0.18

**Table 6:** Characteristics of the study and control groups.

Parameter	Study Group (*n* = 45)		Control Group (*n* = 20)		t/Z	*p* Value
	Mean ± SD	Median (Range)	Mean ± SD	Median (Range)		
Age (years)	40.0 ± 13.9	41.0 (19.0–70.0)	41.4 ± 13.1	42.5 (20.0–60.0)	−0.38	0.71
Diagnosis, *n* (%):			NA		NA	NA
Membranous glomerulonephritis	11 (24.4)					
Minimal change disease	11 (24.4)					
Membranoproliferative	11 (24.4)					
glomerulonephritis						
IgA nephropathy	12 (26.7)					
Type of glomerulonephritis, *n* (%):			NA		NA	NA
Nonproliferative	22 (48.9)					
Proliferative	23 (51.1)					

## Abbreviations

EBV	Epstein-Barr virus
GN	Glomerulonephritis
Ig	Immunoglobulin
NPGN	nonproliferative glomerulonephritis
PD-1	programmed cell death protein 1
PD-L1	programmed death-ligand 1
PGN	proliferative glomerulonephritis
ROC	receiver operating characteristic
